# An interpretable RL framework for pre-deployment modeling in ICU hypotension management

**DOI:** 10.1038/s41746-022-00708-4

**Published:** 2022-11-18

**Authors:** Kristine Zhang, Henry Wang, Jianzhun Du, Brian Chu, Aldo Robles Arévalo, Ryan Kindle, Leo Anthony Celi, Finale Doshi-Velez

**Affiliations:** 1 Harvard University, Cambridge, MA USA; 2 IDMEC, Instituto Superior Técnico - Universidade de Lisboa, NTT DATA Portugal, Lisbon, Portugal; 3 Massachusetts General Hospital, Boston, MA USA; 4 Massachusetts Institute of Technology, Cambridge, MA USA

**Keywords:** Health care, Diagnosis

## Abstract

Computational methods from reinforcement learning have shown promise in inferring treatment strategies for hypotension management and other clinical decision-making challenges. Unfortunately, the resulting models are often difficult for clinicians to interpret, making clinical inspection and validation of these computationally derived strategies challenging in advance of deployment. In this work, we develop a general framework for identifying succinct sets of clinical contexts in which clinicians make very different treatment choices, tracing the effects of those choices, and inferring a set of recommendations for those specific contexts. By focusing on these few key decision points, our framework produces succinct, interpretable treatment strategies that can each be easily visualized and verified by clinical experts. This interrogation process allows clinicians to leverage the model’s use of historical data in tandem with their own expertise to determine which recommendations are worth investigating further e.g. at the bedside. We demonstrate the value of this approach via application to hypotension management in the ICU, an area with critical implications for patient outcomes that lacks data-driven individualized treatment strategies; that said, our framework has broad implications on how to use computational methods to assist with decision-making challenges on a wide range of clinical domains.

## Introduction

Recently, researchers have turned to applying computational methods to analyze historical data for appropriate treatment choices. Reinforcement Learning (RL) is a subset of machine learning in which an agent learns, through observed data, sequences of actions that will maximize some desired outcome. RL methods are particularly well-suited to applications in which outcomes are sparse or delayed, such as patient mortality hours or days after treatments are initiated. The excitement about the potential for RL to assist with healthcare decision-making has lead to applications in managing conditions such as hypotension, cancer, or diabetes^1–5^.

Unfortunately, the computationally learned strategies are usually not human-interpretable^6–8^. This limitation inhibits the ability for clinical experts to inspect and validate a computationally proposed policy prior to deployment. And clinical validation is critical: Prior work has shown that traditional reinforcement learning models tend to be overly optimistic about the benefit of previously untested or under-tested treatments^9^ and conflate clinically distinct conditions together; furthermore, statistical approaches to validation have severe limitations when it comes to identifying potential shortcomings^10^. Recent work has also emphasized the importance of models that provide insight rather than simple prediction^11^.

In this work, we develop a general computational framework to derive easily inspectable treatment policies from historical data. Our core insight is to focus on identifying *decision regions*—similar clinical contexts with respect to patient and disease markers in which clinicians make different treatment decisions—and then using RL to recommend the best option at each “fork in the road.” Not only does this ensure that the RL only suggests options amongst those commonly chosen by clinicians, it results in a small number of easy-to-examine recommendations that can be inspected and validated prior to deployment. (We note that independently of their use for computationally assisted decision-making, these decision regions may also be of intrinsic interest to clinicians curious about situations in which many of their colleagues may be likely to make a different choice than them.)

We demonstrate our framework in the context of managing hypotensive patients in the ICU. Here, sub-optimal choices around fluid and vasopressor administration have been found to dramatically impact the mortality rates of septic patients; furthermore, treatment strategies recommended by standard guidelines are not always consistent with clinician behavior and there is an increasing interest for personalized treatments^12^. For instance, Marik et al. found that the Surviving Sepsis Campaign guidelines recommend a higher level of fluid administration on the first ICU day than is used in practice, and which is associated with increased mortality levels^13^. Bai et al. recommend earlier and more aggressive use of norepinephrine; in contrast, Waechter et al. investigate a lack of understanding of the interaction between fluid and vasopressor treatments, and instead argue that aggressive fluid treatment should be prioritized early rather than vasopressors^14,15^. Given the variety of conflicting evidence and guidelines, there exists a need for more consistent treatment strategies that align with both clinician understanding and data on patient outcomes.

To address this need, a recent set of works have applied RL to identify strategies for managing hypotension in the ICU^1,16,17^, but none have outputs that can be easily checked by clinical experts. In contrast, we provide a strategy that can be interrogated for when recommendations do—and do not—make sense, enabling adjustments prior to any deployment at the bedside. While the policies produced by our approach show promise on various statistical measures of patient outcome, we do not focus on developing a specific treatment policy for bedside use. Instead, we envision these interpretable recommendations being used to elucidate the effects of different treatment options for clinician understanding. This is conducive to further researching and changing the treatment strategy in clinical context where the optimal decision is ambiguous.

## Results

We demonstrate our general computational approach for identifying easily inspected treatment strategies on the question of managing hypotension in the ICU. Our approach was trained and tested using the publicly available Medical Information Mart for Intensive Care version III (MIMIC-III)^18^. After extracting ICU stays involving hypotension symptoms, we obtained a total of 15,653 unique ICU stays. Each ICU stay was encoded as a discrete time series with 1-hour intervals, from the first hour to the 72nd hour since ICU admission. Out of this dataset, 75% of the ICU stays were used for training, and we held out 25% as a test cohort for evaluating learned recommendation performance.

We considered a total of 123 patient condition variables including patient measurements, vital signs, and past treatments received. The list of variables is detailed in the Methods (Data Preprocessing). Because the decision points we identify depend strongly on the feature space used to compare patients, we performed several iterations of the algorithm while incorporating feedback from clinicians. Importantly, the transparency of our approach allowed for feedback to add additional features and focus on patients with mean arterial pressure (MAP) lower than 65 mmHg.

For the purpose of this demonstration, the treatment options are discretized into four unique actions that physicians can take: “no treatment,” “fluids only,” “vasopressors only,” or “fluids and vasopressors both given.” The resulting policy is intended to help clinicians determine which treatment category is best, as there are no existing guidelines beyond the recommendation of initial fluid resuscitation at the time of hypotension presentation. While this discretization is obviously a simplification for the sake of demonstration—we consider neither dosages nor specific drugs—it does capture the core decision; for example, once the choice is made to administer a vasopressor, the dose may be titrated and the choice of drug may be relatively clear based on the patient’s condition.

From these data, we first identify clusters of highly similar patient conditions where patients receive different treatments. We define these areas of high treatment variability as decision regions. Note that we focus only on patients with hypotension, a.k.a. states where patients’ MAP is less than 65mmHg, to focus policy learning on patients who are in the most critical conditions and who are likely to need treatment. We next build a Markov decision process (MDP) that allows decisions only when patients enter decision regions and follows consensus clinical practices for the rest of the times.

**Fig. 1:**
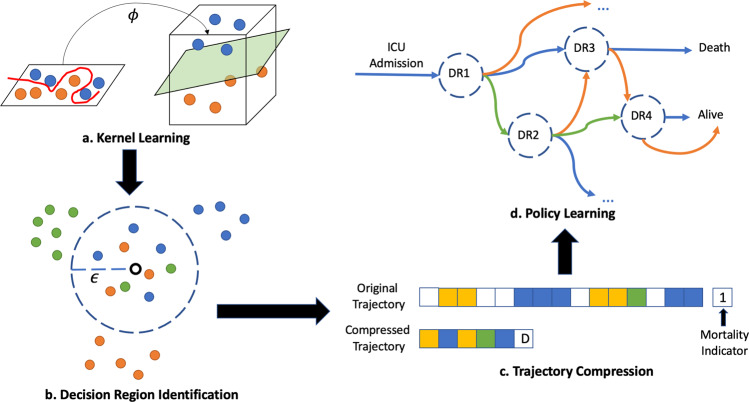
The decision point pipeline. **a** We optimize a similarity metric to reflect physicians' perceptions of patient similarity. **b** We identify decision regions (DR), or areas where similar patients frequently receive different treatments. **c** We summarize patient trajectories in terms of decision regions. **d** We use this Markov Decision Process to learn an optimal treatment policy for each decision region.

Through this process, we are able to filter the 102,844 time points in the data into just 19 decision regions where multiple unique actions were taken for more than 10 patients, allowing us to efficiently propose interpretable treatment recommendations with simple reinforcement learning models for contexts where clinicians disagree. Figure 1 depicts the high-level process of measuring patient similarity, identifying decision regions, building an MDP, and learning policies that can be interpreted and validated prior to deployment.

Three different ways of encoding the goal of managing hypotension were designed to demonstrate that our framework produces treatment strategies that are not only succinct enough to inspect individually, but succinct enough that one can use our framework to surface how different ways of expressing hypotension management goals affect treatment choices. The Methods (Modeling Framework) section provides details on the MDP and reward function formulations. We then performed policy learning to identify treatments that maximize expected returns on the MDP for each reward function. Instead of using deep RL methods to learn policies in a high-dimensional space, simple policy iteration suffices since the number of decision regions is small and tractable. The Methods (Policy Learning) section describes the algorithm in detail. This choice of simple algorithm ensures our policy training always converges to an optimum and allows us to easily explain how each policy was determined.

### Treatment policy recommendations are statistically promising

Table 1 shows that statistical off-policy evaluation (OPE) estimates of the quality of the learned policies (estimated from the historical data) is higher than current practice for every reward function, suggesting potential for improvement. Moreover, the effective sample size (ESS), a measure of confidence that approximates to how much of the data was used to produce the estimate, is over half the size of the dataset. In contrast, other recent attempts to use the same data to identify hypotension treatment recommendations have had ESSs in a few hundred patient trajectories or less^19^. Our higher ESS is a direct result of developing an approach that only attempts to provide recommendations when there appear to be multiple reasonable treatment options for similar patient conditions.

**Table 1:** Off-policy evaluation results of different policies across 5 bootstrap sample runs on the test set.

Policy	WIS Score	ESS
Current Practice	−0.67 ± 0.04	1000 ± 1
MAP-based Rewards	−0.44 ± 0.03	771 ± 6
Mortality-probability Rewards	−0.48 ± 0.03	785 ± 9
Final Survival-based Rewards	−0.30 ± 0.02	705 ± 8

MAP stands for Mean Arterial Pressure. More positive weighted importance sampling (WIS) score indicates better performance. Higher effective sample size (ESS) generally indicates a more reliable estimate. We see a boosted increase on WIS score (0.37 increase for final survival-based policy compared with 0.15 from earlier iteration), a positive sign that iterations using our framework indeed improve the policies.

Specifically, we use a widely used OPE method known as *weighted importance sampling* (WIS)^20,21^. The resulting returns were compared against the average returns obtained by the clinicians’ observed actions. We bootstrapped samples from the holdout test patient trajectories five times and calculated the average rewards. To evaluate the historical treatment strategies of clinicians, we estimate the behavior policy as the empirical proportion of each action taken per decision region. We then average the return over all patient trajectories in the dataset. In addition, we conduct a separate analysis around different policies effects on patients’ lactate levels. The results are positive indications that the recommended actions do potentially improve patients conditions. In addition, we found that these results improved during the final interaction with clinician feedback. Techniques for comparing policy performance are further detailed in Methods (Policy Evaluation).

While WIS is a relatively simple OPE estimator, all OPE estimators have major statistical limitations^10^; promising values are no guarantee that the recommended treatment strategy is effective or even safe. However, the fact that there is some quantitative evidence—in the form of both the value estimate and the ESS—to suggest that these recommended treatment strategies may improve upon current practice provides the motivation to do a thorough human inspection.

### Recommendations are easily inspectable prior to deployment

**Fig. 2:**
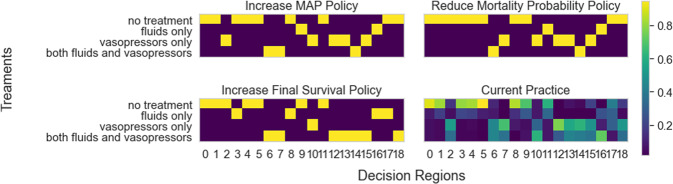
Probability assigned to each action under policies. It is obtained using three different reward functions, compared to the current practices from clinicians.

Figure 2 shows the inferred treatment policy for each of the three ways of codifying the hypotension management goal (known as reward function). In each case, the resulting MDP was solved using standard value iteration to identify a treatment policy with the highest expected rewards. Being able to use simple, robust optimization methods is an advantage our method enjoys over having to use deep RL methods to learn treatment policies in a high-dimensional spaces. Unlike treatment policies derived via deep RL, where it is not possible to display even a single proposed treatment policy, here we can show policies learned from all three reward functions as well as the clinician’s current practice for reference all in one figure for inspection and critique.

**Fig. 3:**
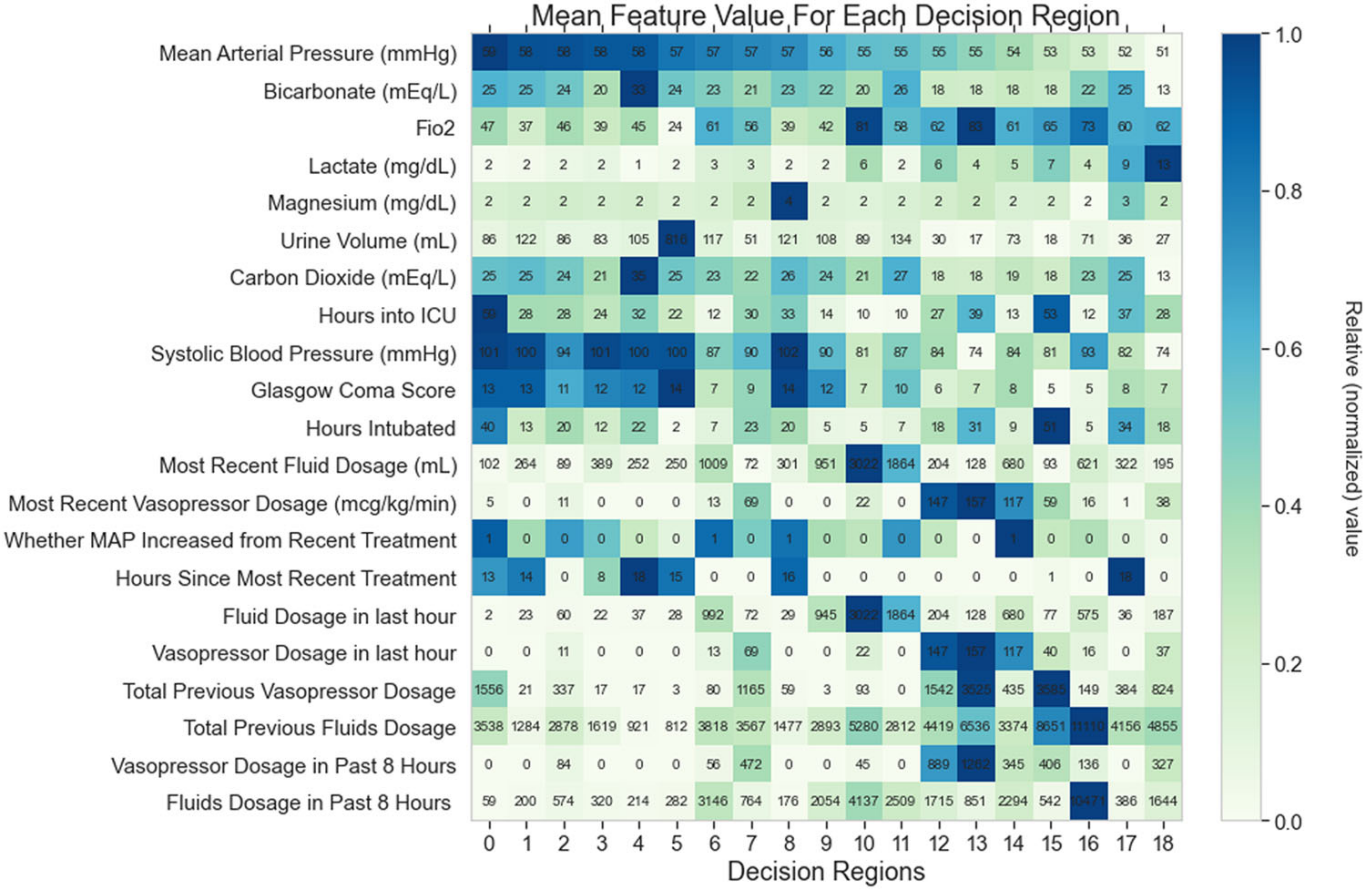
The feature means for points in each decision region. Means are sorted by descending order of Mean Arterial Pressure (MAP) value.

**Fig. 4:**
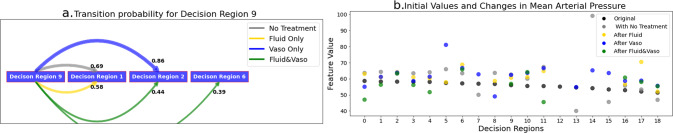
Change in patient state after different treatments. **a** The probability of patients moving from one decision region to other decision regions when given different treatments. This example figure shows how patients transition out of decision region 9. **b** The expected average feature value for patients in each decision region after different treatments were given. This example figure reflects MAP level change.

For the inspection of the treatment strategies below, we also provide the visualizations in Figs. 3, 4. The first summarizes the mean values of key clinical measures in each decision region; the others show the expected change in each of these measures under each treatment and the expected transitions between decision regions after each treatment.

We specifically focus on decision regions with high mortality rate (≥10%) and patient counts (≥10 patients each for at least two different treatments). By examining these plots in conjunction with the recommendations in Fig. 2, clinicians can map MDP states to their practical experiences. They can also reason about whether the generated policy is safe and justifiable. Again, the succinctness of these visualizations enables expert interrogation of each suggestion in each decision region. Such detailed clinical feedback is critical for understanding what types of patients a decision region represents and verifying whether the policy recommendation is reasonable prior to any deployment. Discrepancies between clinician opinions and the learned policies can then be further investigated. This interrogation of learned RL policies is enabled by our framework and would not be possible with a black-box policy.

### Inspection of the recommendations yields useful insights

We now perform the interrogation of our computationally learned treatment policies. Importantly, we do not claim that our learned policies are correct. Rather, our core contribution is that the policies can be inspected and validated. Below, we describe instances where this ability to inspect the policies allowed us to identify situations in which the recommended treatment strategies made clinical sense—and where they did not.

Firstly, we note the fact that policies based on different reward functions are slightly different but largely similar, especially with regards to the decision regions where treatment is recommended, demonstrates the recommendations are most robust to choices of exactly how the hypotension management goal is formalized to the RL algorithm.

More specifically, many of the recommendations make sense. The learned policies and current practice both are less likely to recommend treatments for lower-numbered decision regions which correspond to patient states with higher average MAP. This makes sense as patients in those conditions are relatively stable. In general, the treatments learned by RL models are slightly more conservative than the current clinicians’ policy. While “vasopressor only” or “vasopressor and fluids” are the most popular action in 10 of 19 decision regions, our learned policies tend to recommend these relatively aggressive actions in only 7 or 8 decision regions. This is clinically plausible given recent studies have found fluids may be overused and potentially worsen outcomes in the ICU^22^. This trend also reflects the “less is more” mentality regarding treatments in the ICU that has gained traction over the last decade^23^.

In particular, we highlight a couple decision regions where our learned policy diverges from the clinicians’ observed policy. In decision region 2, the MAP level suggests only borderline hypotension and both the “mortality probability” and “outcome-based” policies suggest action 0 (no treatment); however, the “increase MAP” policy agrees with the physicians’ tendency to choose action 3 (just vasopressor) or action 4 (fluid and vasopressor). From the feature matrix in Fig. 3, most patient metrics including lactate, urine, FiO2, and systolic blood pressure do not appear concerning. It appears that clinicians may be reacting mostly to the borderline hypotension and attempting to increase MAP via vasopressor use, but our algorithm suggests that a more conservative approach may be reasonable given the normality of the patients’ statistics aside from MAP.

Decision regions 6 and 7 are both cases in which clinicians tend to choose between vasopressors only or vasopressors and fluids, but they are more likely to use vasopressors only in decision region 7. Here, the recommended policy tends to be more aggressive than the clinicians and recommends using both vasopressors and fluids. Patients in region 6 have already received high recent fluid and vasopressor dosages, and have high FiO2 levels, so clinicians may hesitate to give fluids to avoid fluids entering their lungs. Similarly, patients in state 7 have already received high vasopressor dosages, so clinicians may hesitate to continue giving vasopressors. The recommended treatment policy of both fluids and vasopressors suggests the computer does not consider the high previous dosages to be as prohibitive as clinicians do, which prompts further investigation of similar cases.

Decision regions 17 and 18, which contain patients with the most severe hypotension, also prompt further investigation. In these cases, both the "increase MAP” and "reduce mortality” learned policies recommend taking no action, though physicians are split between different options. With such low MAP and high lactate, doctors may not see a way of saving the patient, and the algorithm learns that there is little benefit in giving treatments. Both the algorithm’s and clinicians’ policies may be attributed to the severity of these cases–any treatment, including no treatment, likely leads to a poor outcome. Clearly, the question of whether to treat in the most futile conditions is worth more discussion. At the same time, it signifies the importance and necessity of a clinical tool like ours that can be thoroughly interrogated while making a treatment decision.

**Fig. 5:**
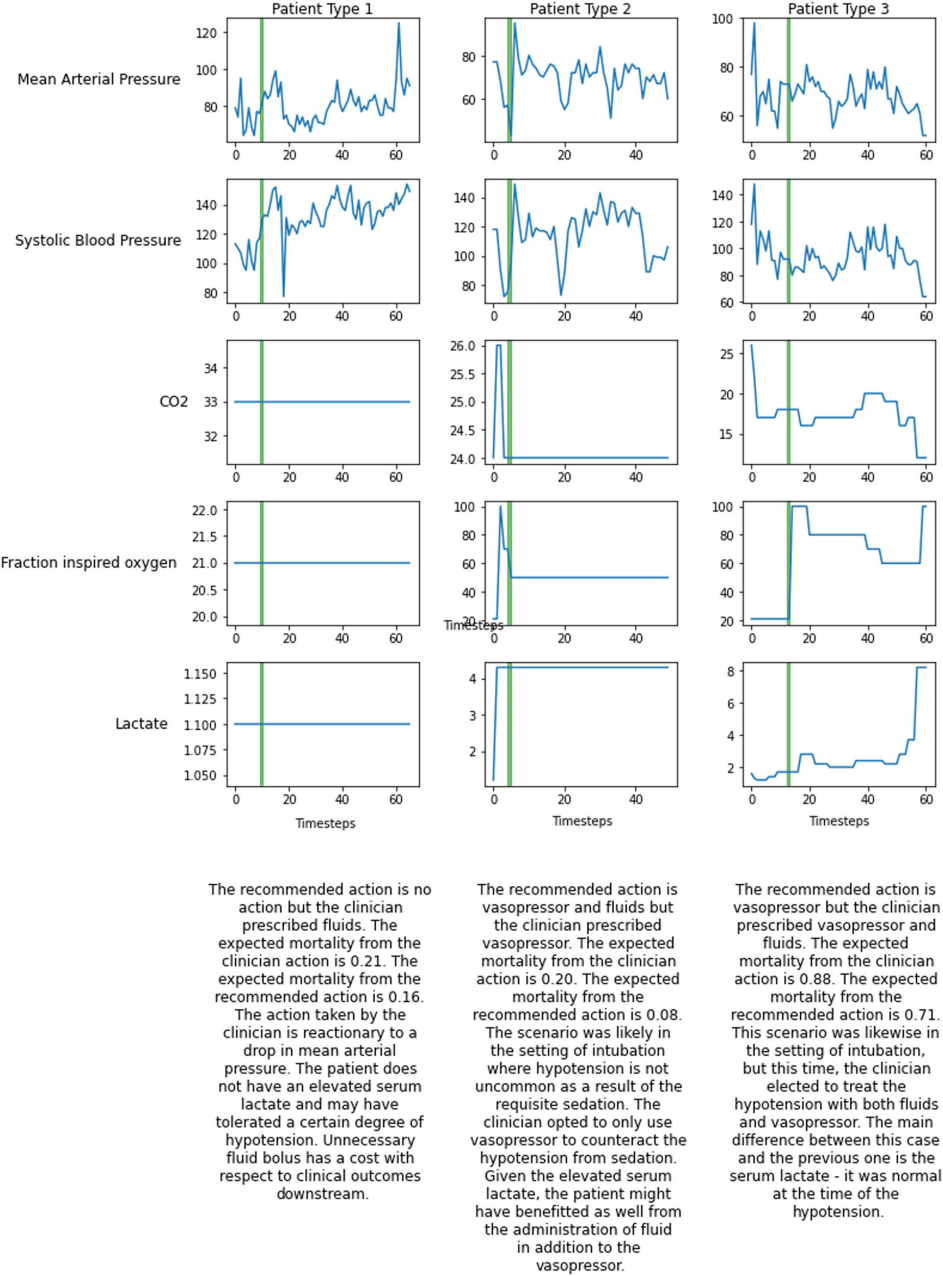
Examples of patient trajectories. For three specific patient cases where the recommended action differs from the clinician actions, we track the changes over time in their clinical metrics.

Specific examples of the effect of recommended action can be found in Fig. 5. Several different patients are shown where the recommended action differs from the action taken by the clinician. Patient type 1 shows an example where the recommended action is to take no action, but the clinician prescribed some treatment. The expected mortality from the recommended action is lower than the clinician action. The action taken by the clinician is reactionary to a drop in mean arterial pressure. The patient does not have an elevated serum lactate and may have tolerated a certain degree of hypotension. Unnecessary fluid bolus has a cost with respect to clinical outcomes downstream. Patient type 2 is an example where the algorithm recommends a treatment different from the clinician treatment that results in a lower expected mortality. The scenario was likely in the setting of intubation where hypotension is not uncommon as a result of the requisite sedation. The clinician opted to only use vasopressor to counteract the hypotension from sedation. Given the elevated serum lactate, the patient might have benefitted as well from the administration of fluid in addition to the vasopressor. Patient type 3 shows an example where the expected mortality for either the recommended and clinician action is high. This scenario was likewise in the setting of intubation, but this time, the clinician elected to treat the hypotension with both fluids and vasopressor. The main difference between this case and the previous one is the serum lactate; it was normal at the time of the hypotension.

## Discussion

We leverage our general framework for interpretable RL to derive recommendations for managing hypotension in the ICU from historical data. The resulting treatment guidelines not only have promising statistical performance, but can also be inspected by experts to identify specific recommendations for further study. In this way, our approach enables much more in-depth analysis leveraging historical data: we produce a collection of easily inspectable treatment suggestions in contexts where there is no clinician consensus that can be inspected and validated prior to any deployment at the bedside. We have also demonstrated the value of increased transparency through improved policies by iterating over clinician feedback. In contrast, many existing black-box RL systems provide specific treatment suggestions for individual patients, but are difficult for clinicians to understand and learn from at a high level.

In this way, we take important steps toward addressing a critical gap in the safe and effective adoption of reinforcement learning algorithms at the bedside. The ability to interrogate the model as we demonstrated in this paper will help clinicians understand: (1) how a specific episode of hypotension given a certain set of features was treated historically and (2) identify the intervention that typically resulted in the best outcome. Rather than relying on a black-box model to make decisions in real time, clinicians can use the interpretable model suggestions beforehand to identify the most contentious patient contexts and re-examine or modify their treatment strategies. Describing the model suggestions in terms of a small number of decision regions allows clinicians to focus on and visualize the most complex patient contexts, and also identify possible weaknesses in the model, such as clustering clinically distinct patients together. A secondary use case would be to alert clinicians when the algorithm detects a patient entering a decision region. The clinician would then have the option to compare their intended treatment against their colleagues’ past choices and/or the model’s recommendation.

More broadly, our method may be used to identify safe and interpretable recommendations on many clinical problems beyond hypotension management in critical care. Though we have shown our approach’s applicability for this use case, we present a method, rather than a specific model, that can summarize reinforcement learning models for better clinician understanding. RL is a useful tool when one wishes to utilize large amounts of historical data to enhance the clinician’s prediction of potential long-term effects, perceived short-term gains (e.g. immediate physiologic improvement) from specific actions, and treatment interactions that are not typically investigated in clinical trials. That said, in the midst of treating patients, clinicians may not and should not be willing to blindly rely on automated recommendations in complex contexts without inspecting the historical data that supports a given choice. In contrast to previous RL approaches that are black-box, our model’s recommendations are highly interpretable and enable expert-guided policy improvement. Because our recommendations are so succinct, following expert validation and further prospective testing, they can be summarized into guidelines *that require no computer at the bedside*. Thus, we have a natural pathway from inspection, adaptation, prospective validation, and integration into practice.

With regard to the specific question of hypotension management, our approach is an important step forward but has its limitations. Due to our focus on presenting the framework that outputs explainable recommendations, we generated recommendations of fluid or vasopressor without doses because that is what was supported by the data available. With regard to the outcome: while 30-day mortality is an easily measurable outcome, there are many factors contributing to that outcome beyond how the patient’s hypotension was managed. Finally, our methods take several measures to avoid confounding as detailed in Methods (Modeling Framework), but it is still quite possible that certain decision regions do have unmeasured confounders.

Relatedly, one inherent challenge in using data retrospectively to evaluate models is the lack of clarity into the original physicians’ decision processes. Our analysis allowed us to inspect specific cases where physicians’ observed actions differed from our algorithm’s recommendation. Aiming to understand the clinical reasoning behind the original actions, we identified 50 such patients and reviewed clinician progress notes around the times of disagreement. These notes stated general plans of action such as “wean vasopressor as tolerated,” “optimize preload,” or “trend serum lactate,” but did not include any rationale for specific actions. Because the underlying data does not capture the physicians’ reasoning, it is difficult to identify the cause of model divergences. Instead, a retrospective study relies on other clinicians to examine these specific cases and propose potential explanations.

To these limitations, we emphasize that our goal was not to provide the unequivocal solution to hypotension management in the ICU, but rather provide a demonstration of a method that, through its inspectability, enables a conversation about what recommendations are worth further investigation and those that are likely caused by confounders or errors in data capture—something impossible with current RL methods.

More broadly, our framework does involve multiple computational steps that must be tuned depending on the dataset, including iteratively updating the clusters to select the best set of hyperparameters. We tested robustness to one key set of parameters—the rewards—in this work, and validated other parameters via iterative tuning and clinical expertise. If one were to apply our framework to a new clinical domain, all of this tuning would need to be done to ensure valid results; an interesting direction for future computational work could include systematic methods for automating this turning or reducing the number of moving parts.

We introduced a framework for investigating contexts in which clinicians choose different treatments for similar patients, identifying the better options in these cases, and succinctly presenting this analysis back to clinical experts. In the application of hypotension management in the ICU, we presented 4 different treatment options across patient states as defined by vital signs, laboratory test results, and treatment history - no treatment, fluids only, vasopressors only and fluids plus vasopressors - and their associated clinical outcomes. The concise treatment suggestions produced by our model had some quantitative evidence for improving care, but more importantly, they enabled visualizations that clinicians may use to verify whether gains could truly be expected.

As we have done, we can continue updating our framework to integrate new data and input from human experts into the pipeline (i.e., human in the loop) for iterative policy improvement and customized treatment plans for patients in the future. Furthermore, the overall approach can be validated by testing the framework on new datasets and determining whether the resulting suggestions are clinically sound. Reinforcement learning models have shown great promise thus far for medical applications; our framework aims to help clinicians fully leverage their value using clinician-tested and interpretable treatment recommendations.

## Methods

### Dataset

We used data from the Medical Information Mart for Intensive Care (MIMIC-III) dataset^18^. The dataset contains charted observations and laboratory measurements for patients who were treated in the intensive care units at Beth Israel Deaconess Medical Center. MIMIC-III is available at http://mimic.physionet.org/. We used data from the MIMIC-III MetaVision database, which includes stays from 2008 and onwards. Our inclusion criteria were that it was the first ICU stay for that patient and the stay had at least 3 MAP measurements below 65 mmHg, indicating hypotension. The filtering resulted in a total of 15,653 unique ICU stays.

### Data Preprocessing

Preprocessing was performed as described in Futoma et al.^19^. Each ICU stay was considered a single trajectory. Trajectories were discretized into hourly bins starting at *t* = 1 h into ICU admission and truncated either at discharge or at *t* = 72 h, as most acute hypotensive cases tend to appear early in ICU admission. We applied the “last observation carried forward” (LOCF) method for imputation - If no measurements or multiple measurements were taken in a given hour, the most valid recent measurement was used. Because many lab measurements happen once in several hours, this method provided us conservative estimate for missing values without introducing additional noises. It is also uncommon for lab values to be measured multiple times within an hour and many vitals are measured only once per hour for MIMIC-III so we rarely lost measurements inside the hour^19^.

The initial state space consisted of 123 clinical features including patient measurements, vital signs, and records of past treatment actions. In addition to those features, we also created indicator variables to denote how recently the measurements were taken—in the past hour, in the past 8 hours, or any time during the ICU stay. We also included past treatment features including the amount of either fluids or vasopressors administered to each patient at each time step. The complete list of features considered can be found in Table 2; note these dimensions contain some redundancy with indicators and quantitative measure for the same variables.

**Table 2:** List of clinical features considered from the MIMIC-III dataset.

Category	Features
Static/Demographic	Admission Times
	Admission Times (normalized)
	Age
	Gender
	If Surgical ICU
	If Ethnically White
	If Emergency Admission
	If Urgent Admission
	Hours since Hospital Admission to ICU Admission
Labs Measurements	Bicarbonate
	Bun
	Creatinine
	Fio2 (fraction inspired oxygen)
	Glucose
	HCT (hematocrit)
	Lactate
	Magnesium
	Platelets
	Potassium
	Sodium
	White Blood Cell Count
	Alt: liver marker
	Ast: liver marker
	Bilirubin: liver marker
	Hemoglobin
	pco2:partial pressure of carbon dioxide
	po2:partial pressure of oxygen
	CO_2_
	Weight
Vitals	Heart Rate
	Pulse Oximetry
	Temperature
	Urine Output
	Respiration Rate
	Diastolic Blood Pressure
	Mean Arterial Pressure
	Systolic Blood Pressure
	Glasgow Coma Score

In policy learning we primarily consider the four general actions corresponding to “no treatment” (83% of the time steps in the cohort), “fluids only” (3%), “vasopressors only” (12%), or “fluids and vasopressors both given” (2%). The learned policy is intended to help clinicians decide whether to give each type of treatment at all, as there are existing guidelines for the quantity to give once a choice has been made.

To analyze treatment impact on outcome, we define a patient’s mortality to be true if they died in the hospital within 30 days of ICU admission, which accounts for 11% of the patients in the dataset.

For optimization, we consider three different reward functions, or ways to quantify outcomes: (1) Outcome-based: the patient is only given a reward once they reach one of two mortality states at the end of the trajectory. The reward is 1 for reaching “alive” and 0 for reaching “dead”, 30 days after ICU admission. (2) Mortality-probability: at each timestamp, the reward is calculated based on the current cluster the patient is in and the treatment given. The reward is the negative empirical probability of mortality for the cluster-treatment combination. There is 0 reward for leaving a mortality cluster. (3) Mean Arterial Pressure-based: at each timestamp, the reward is calculated based on the average MAP of the next cluster the patient enters. Lower MAP corresponds to lower reward, with the reward calculated as a linear interpolation from −1 to 0. For mortality states, the reward of “alive” is 0 and the reward of “dead” is −1.

While this first reward corresponds most closely to what we may care about, it is also distant (occurring a long time after the treatment action), and there may be many other reasons for the mortality outcome. The probability of mortality reward function makes the distant outcome more immediate, and the MAP-based reward function helps focus on elements that the treatments most directly affect.

### Modeling framework

Formally, a Markov Decision Process (MDP) is a tuple $\langle {{{\mathcal{X}}}},{{{\mathcal{A}}}},{{{\mathcal{T}}}},{{{\mathcal{R}}}},\gamma \rangle$. The state space ${{{\mathcal{X}}}}$ can be discrete or continuous ($\in {{\mathbb{R}}}^{d}$). We shall assume a discrete action space ${{{\mathcal{A}}}}$. The functions $T(x^{\prime} | x,a)$ and *R*(*x*, *a*) denote the state transition function and reward function, respectively; the discount factor *γ* ∈ (0, 1] trades between immediate and longer-term outcomes.

A policy $\pi :({{{\mathcal{X}}}},{{{\mathcal{A}}}})\to [0,1]$ defines the probability an action given a state. We define *π*_*b*_ as the behavior policy under which the observed data was generated, in this case the current clinicians’ policy, and *π*_*e*_ as the evaluation policy learned through RL models. The objective is to learn a policy that maximizes the expected return, or sum of discounted rewards ${V}^{\pi }=E\left[{\sum }_{t}{\gamma }^{t}{r}_{t}| {a}_{t} \sim \pi \right]$.

We split the set of patient trajectories into train and test sets. We used a 75/25 split resulting in 11,739 trajectories in the train set and 3914 trajectories in the test set. We then select records where patients’ mean arterial pressure is below 65mmHg as those are likely moments where patients are in the critical conditions. This resulted in 102,844 transition tuples for the train set and 34,549 transition tuples for the test set.

To generate an MDP from these transitions, we first identified a set of decision regions, or patient conditions under which clinician disagreement is most common. The innovation of our method is that we do not consider every time-step to be a potential decision point. Rather, we only consider those points with high behavior policy variability, that is, states whose similar neighbors are administered different treatments.

Figure 1 outlines the pipeline used to condense initial patient states into decision regions. We first apply kernel learning to learn the *similarity* among patients in a latent space. We then identify areas where similar patients frequently receive different treatments, a.k.a. *Decision Regions* (DR). Next, we summarize the trajectories in terms of decision regions. We finally use this Markov Decision Process to learn an optimal policy over the decision regions. Each of these steps are detailed below.

**Fig. 6:**
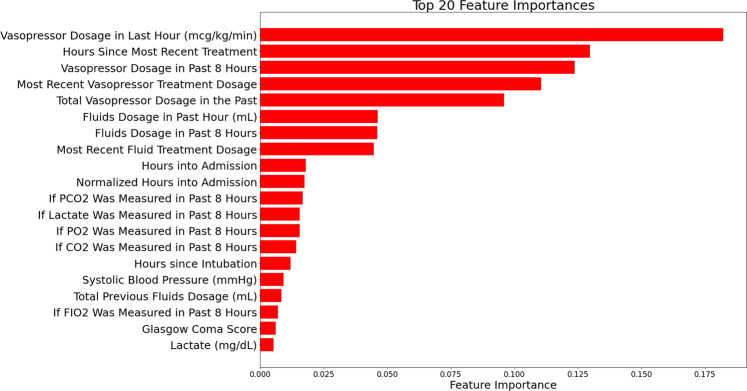
Feature importance of clinical variables. Bar charts showing feature importance, derived from random forest classifiers, in descending order.

To identify decision points, we train a Random Forest classifier to identify the top 20 patient features with the highest importance score for predicting treatment actions. Figure 6 shows these 20 variables. We then combine those classifier-identified features with features that clinicians believe are important, such as indicator of whether CO_2_ is within 21–29 mEq/L. Afterwards, we train a kernel-based classifier to predict clinician actions, simultaneously learning a weighted Gaussian kernel as a distance metric between states.

1 $$k(x,x^{\prime} )=\exp (-\parallel ({{{\boldsymbol{w}}}}\odot x-{{{\boldsymbol{w}}}}\odot x^{\prime} ){\parallel }_{2}^{2})$$

Here, $k(x,x^{\prime} )$ is the estimated similarity between states, ***w*** can be interpreted as an importance weighting over state dimensions, and ⊙ represents an element-wise multiplication between vectors. We learn the kernel weights ***w*** by backpropagating through the kernel space to optimize the cross-entropy loss of predicting clinician actions. We use a Random Fourier Feature^24^ representation of the kernel for additional computational scalability. States whose close neighbors (according to the kernel) disagree on the choice of action are considered *decision points*. States with kernel similarity threshold of 0.95 and greater are considered as neighbors, and an action with at least 20 neighboring patients taking is considered choosable.

After identifying whether each state is a decision point, we proceed to cluster the decision points into *decision regions*. We utilize a top-down hierarchical clustering approach where all decision points start in the same cluster and clusters are iteratively split. Distances between decision points are calculated using the standardized state features (dimensions of *x*). For each intermediate cluster, the difference of mean values for each feature across actions is computed. If the differences in means exceed 1, this implies the region is not homogeneous and we further split the clusters. This ensures finding decision regions where the optimal action is truly unclear. Secondly, we examine if a given cluster creates loops—defined as trajectories that leave a cluster and return to the same cluster within the next three time steps—as these (artificial) loops can make the agent believe that it can “freeze time” and avoid any final consequence. If loop percentage is over 20%, we further split the cluster. This clustering serves as the starting point for us to define our summarized MDP. Each *decision region* is now an *state* in our summarized MDP.

The clusters form the states of our summarized MDP. It remains to specify how we treat actions and define the transitions between the decision regions. Our first step is to condense contiguous times when a patient is in a specific decision region as one visit to that decision region. The action for a single decision region is defined as the combination of any treatments given during the recorded time period. For example, a patient who receives no treatment throughout their time in a decision region will be designated “no treatment”, whereas a patient who receives fluids and then vasopressors while staying in the same decision region will be designated “fluid and vaso”.

Next, we remove any time steps in the patient trajectory that are not decision clusters (that is, non-decision point states). In the final tuple of a trajectory, we include a transition to the mortality state of the patient to track their outcome. In mathematical terms, we convert each patient transition tuple ${\{(x,a,x^{\prime} )\}}^{L}$ into a shorter trajectory ${\{(\bar{x},\bar{a},\bar{x}^{\prime} )\}}^{{{{\mathcal{\ell }}}}}$, where all $\bar{x},\bar{x}^{\prime} \in {{{\mathcal{C}}}}$ are decision clusters and $\bar{a}\in \bar{{{{\mathcal{A}}}}}$ are summarized actions. This gives us a new dataset with a total $\bar{N}$ transition tuples $\{(\bar{x},\bar{a},\bar{x}^{\prime} )\}$. Now, we estimate a behavior policy ${\bar{\pi }}_{b}$ and transition function $\bar{T}(\bar{x}^{\prime} | \bar{x},\bar{a})$ by iterating over the new dataset, as formalized in Equation (2) and (3). Intuitively, the behavior policy is estimated as the proportion of times an action was taken from a given decision cluster, and the transition function is estimated as the proportion of times a given decision cluster-action pair led to another decision cluster. This MDP summarization process reduces both the size of the state space under consideration and the length of patient trajectories.

2 $${\bar{\pi }}_{b}({\bar{a}}^{* }| {\bar{x}}^{* })=\frac{\mathop{\sum }\nolimits_{i = 1}^{\bar{N}}{\mathbb{I}}({\bar{x}}_{i}={\bar{x}}^{* },\,{\bar{a}}_{i}={\bar{a}}^{* })}{\mathop{\sum }\nolimits_{i = 1}^{\bar{N}}{\mathbb{I}}({\bar{x}}_{i}={\bar{x}}^{* })}$$

3 $$\bar{T}(\bar{x}{^{\prime} }^{*}| {\bar{x}}^{*},{\bar{a}}^{*})=\frac{\mathop{\sum}\nolimits_{i = 1}^{\bar{N}}{\mathbb{I}}({\bar{x}}_{i}={\bar{x}}^{*},\,{\bar{a}}_{i}={\bar{a}}^{*},\,\bar{x}^{\prime} =\bar{x}{^{\prime} }^{*})}{\mathop{\sum}\nolimits_{i = 1}^{\bar{N}}{\mathbb{I}}({\bar{x}}_{i}={\bar{x}}^{*},\,{\bar{a}}_{i}={\bar{a}}^{*})}$$

When identifying decision regions we have two main hyperparameters: *δ*, the minimum similarity from a point *x* to its neighbors, and *n*, the minimum number of neighbors who must share the same action. Any action taken by at least *n* neighbors within similarity *δ* are considered allowable actions. Decision points are defined as states with multiple allowable actions in their neighborhoods. To choose the optimal hyperparameters, we perform a grid search over a set of candidate values on a validation dataset. For each pair of *δ*, *n*, we evaluate the overall AUC score from action classification for a sampled subset of points. The labels are determined by the empirical distribution of observed actions in each neighborhood. Through gridsearch, we finally choose the similarity threshold *δ* = 0.95 and *n* = 20, which achieves the highest AUC score.

To check for sensitivity to reward formulation, we solved our MDP using three different reward functions. (1) MAP-based Rewards: at each timestamp, the reward is calculated based on the average MAP of the next cluster the patient enters. Lower MAP corresponds to lower reward, with the reward calculated as a linear interpolation from −1 to 0. For mortality states, the reward of “alive” is 0 and the reward of “dead” is −1. (2) Mortality-probability Rewards: at each timestamp, the reward is calculated based on the current cluster the patient is in and the treatment given. The reward is the negative empirical probability of mortality for the cluster-treatment combination. There is 0 reward for leaving a mortality cluster. (3) Outcome-based Rewards : the patient is only given a reward once they reach one of two mortality states at the end of the trajectory. The reward is 1 for reaching “alive” and 0 for reaching “dead”, 30 days after ICU admission.

### Policy Learning

Given the summarized MDP above, we solved for its optimal policy using value iteration, which computes the expected reward associated with a state. Value iteration begins by initializing the expected reward of each state to random values and proceeds by iteratively updating the value function until it finds a policy that maximizes long-term expected rewards. The value *V* of state *s* is updated using Bellman’s equation:

4 $$V(x)\leftarrow \mathop{\max }\limits_{a\in {{{\mathcal{A}}}}}\left(E[r| x,a]+\gamma \mathop{\sum}\limits_{x^{\prime} \in X}P(x^{\prime} | x,a)V(x^{\prime} )\right)$$

Because we constructed three different reward functions, we learned three corresponding policies. Because the rewards are sparse, we used discount rate of *γ* = 0.98 to avoid neglecting future rewards. We tried discounts of 0.95 and 0.9 in earlier iterations and the optimal choices of treatments were consistent, indicating our policies were robust to the choice.

### Policy Evaluation

To quantitatively evaluate the optimal policy learned using the designed MDP, we employ techniques from off-policy evaluation (OPE). OPE can be used to estimate the value ${V}^{{\pi }_{e}}$ of an evaluation policy *π*_*e*_ given only historical trajectories ${{{\mathcal{D}}}}=\{{\tau }^{(1)},\ldots ,{\tau }^{(n)}\}$ collected with a behavior policy *π*_*b*_.

The most common approach to OPE is *importance sampling* (IS). The vanilla IS estimator is given by ${\hat{V}}_{IS}^{{\pi }_{e}}({{{\mathcal{D}}}})=\frac{1}{n}\mathop{\sum }\nolimits_{i = 1}^{n}g({\tau }^{(i)}){\rho }^{(i)}$ where the trajectory weight is a product of likelihood ratios ${\rho }^{(i)}=\mathop{\prod }\nolimits_{t = 1}^{{T}^{(i)}}\frac{{\pi }_{e}({a}_{t}^{(i)}| {s}_{t}^{(i)})}{{\pi }_{b}({a}_{t}^{(i)}| {s}_{t}^{(i)})}$ and $g(\tau )=\mathop{\sum }\nolimits_{i = t}^{T}{\gamma }^{t}R({s}_{t},{a}_{t})$ as the discounted return of a trajectory. Unlike the trajectories that are typically used in the IS estimator, our decision region trajectories have varying lengths *T*^(*i*)^. Although the IS estimator is unbiased for ${V}^{{\pi }_{e}}$, it usually has large variance. To reduce variance, the *weighted importance sampling* (WIS) estimator is more widely used as a biased but consistent estimator. The WIS estimator normalizes the importance weights *ρ* of each trajectory within range 0 to 1. We might prefer WIS to IS when there are few samples available, because the lower variance of WIS is able to produce a larger reduction in expected square error than the additional error incurred due to the bias.

5 $${\hat{V}}_{WIS}^{{\pi }_{e}}({{{\mathcal{D}}}})=\frac{1}{\mathop{\sum }\nolimits_{i = 1}^{n}{\rho }^{(i)}}\mathop{\sum }\limits_{i=1}^{n}g({\tau }^{(i)}){\rho }^{(i)}$$

We utilize the WIS estimator in our experiments and apply standard weight clipping by capping weights at 95 percentile. We construct evaluation rewards by combining MAP-based rewards and Mortality-probability rewards - reward based on MAP value during ICU stay and mortality-probability reward at ICU release. Specifically, during the ICU stay we assign −1 to 0 reward to patients based on the MAP readings for each step and at the end of the trajectory, assign −1 if they reach “death” and 0 if they reach “alive”. For each of our learned policy as well as current practice policy (historical treatment records from clinicians), we apply WIS over all trajectories and take the average WIS score as the estimate of a policy’s value. For behavior policy, we use the percentage of each action taken in historical treatments as the probability estimate of taking the corresponding action and thus calculate the behavior policy’s WIS score.

To provide additional quantitative support, we evaluate the effects of different policies on the transition of patients’ lactate level as a proxy for whether the treatments are effective. For hypotensive patients, reduction in lactate level suggests treatment efficacy. For each occurrence of lactate change, we record the provided treatment given the hour before and determine whether the treatment provided by clinicians are consistent with the recommendations provided by our policies. We then apply WIS to estimate the reduction in lactate were our recommended actions were provided. While our policies were not specifically trained to reduce lactate, we find our policies achieve similar performance in reducing lactate as clinician policy. We achieve WIS estimated reduction of 0.06 for behavior policy and 0.08 for evaluation policy with MAP-based rewards and final survival-based rewards on the test set, which is positive sign that the recommended actions are improving the patients’ conditions.

### Reporting summary

Further information on research design is available in the Nature Research Reporting Summary linked to this article.

## Supplementary information

Reporting Summary

Supplemental Material

## Data Availability

The hypotension data that support the findings of this study are available in MIMIC-III with the identifier doi:10.1038/sdata.2016.35. MIMIC-III is openly available.

## Code availability

The code that produces the results in this manuscript can be accessed at https://github.com/dtak/Decision-Region-for-ICU-Hypotension, which includes detailed insturctions for running the code. Python 3.7, PyTorch 1.10, Numpy 1.21, Sklearn 1.0.1 and Pandas 1.3.4 were used.
