# Supplementary material for: An interpretable RL framework for pre-deployment modeling in ICU hypotension management
Source: NPJ Digit Med. 2022 Nov 18;5:173. doi: 10.1038/s41746-022-00708-4 (PMC9671896; doi:10.1038/s41746-022-00708-4)
Supplement: Supplementary file 2 — Supplemental Material [file 41746_2022_708_MOESM2_ESM.pdf]

Supplementary Information

In the latest iteration, we only consider states where patients' mean arterial pressure (MAP) is below 65mmHg. We incorporated features that clinicians refer to when making treatment decisions during the kernal training for decision points. The results from the previous model iteration are included below.

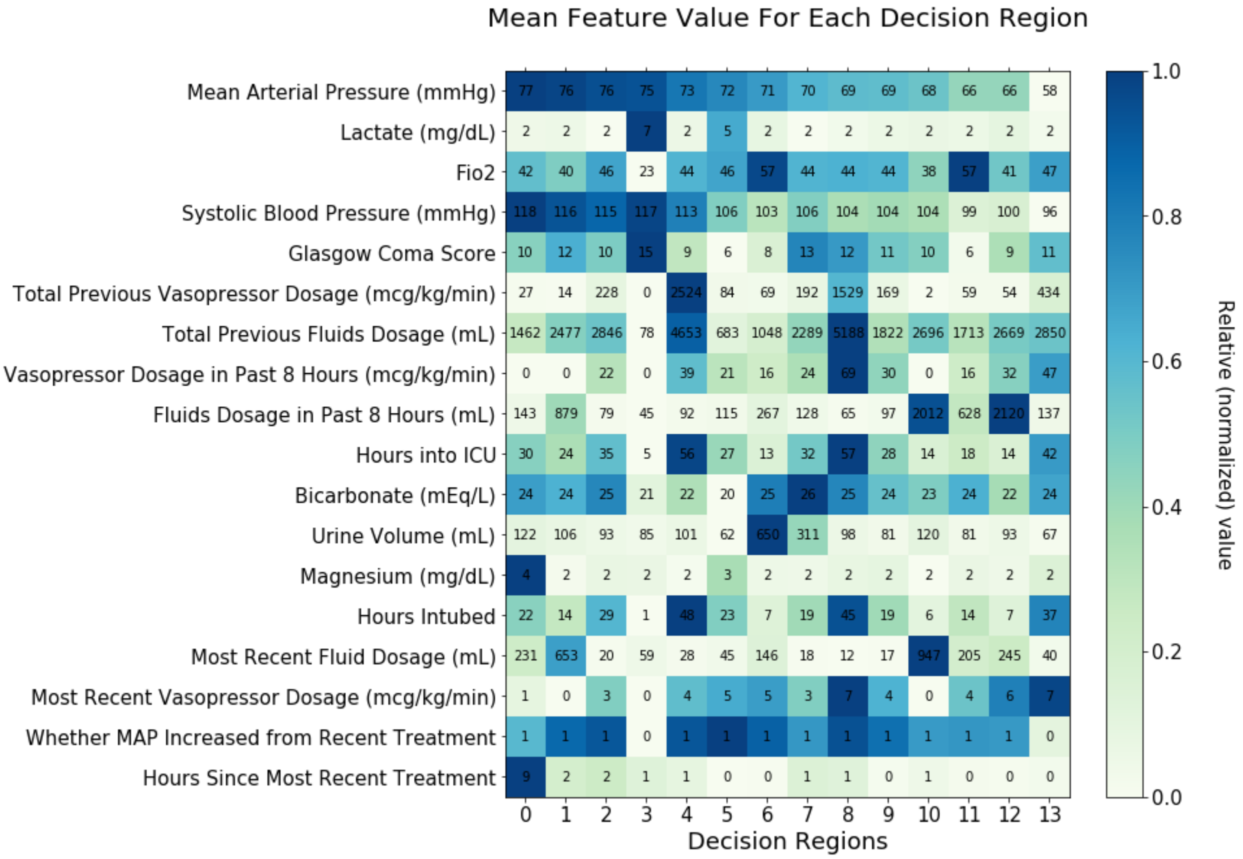

Supplementary Figure 1: **Feature Means for Decision Regions.** Previous results. The feature means for points in each decision region. Sorted by descending order of Mean Arterial Pressure (MAP) value.

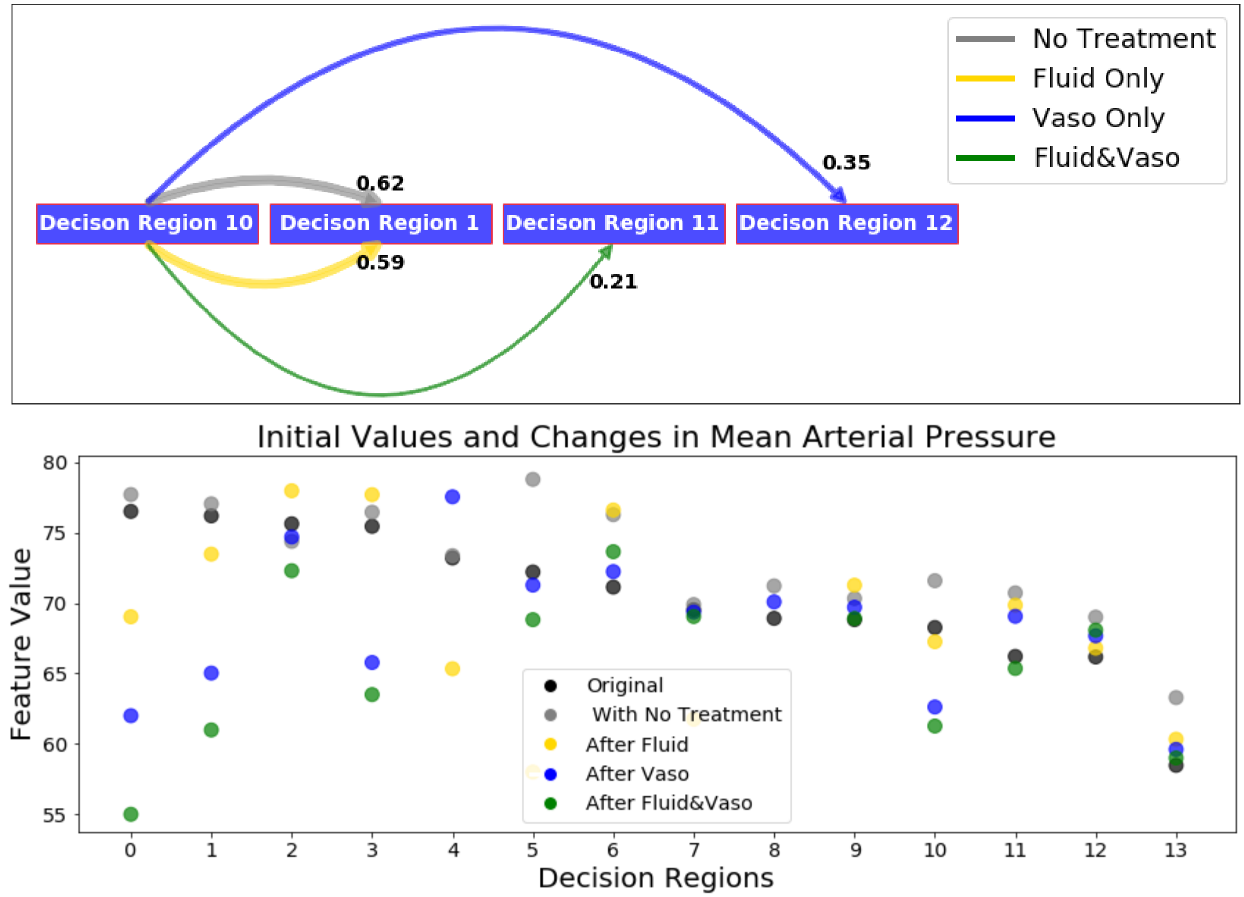

Supplementary Figure 2: **States Change after Treatment.** Previous results. Change in patient state after different treatments. Top figure is probability of patients moving from one decision region to other decision regions when given different treatments. This example figure shows how patients transition out of decision region 10. Bottom figure is expected average feature value for patients in each decision region after different treatments were given. This example figure reflects Mean Arterial Pressure level change.

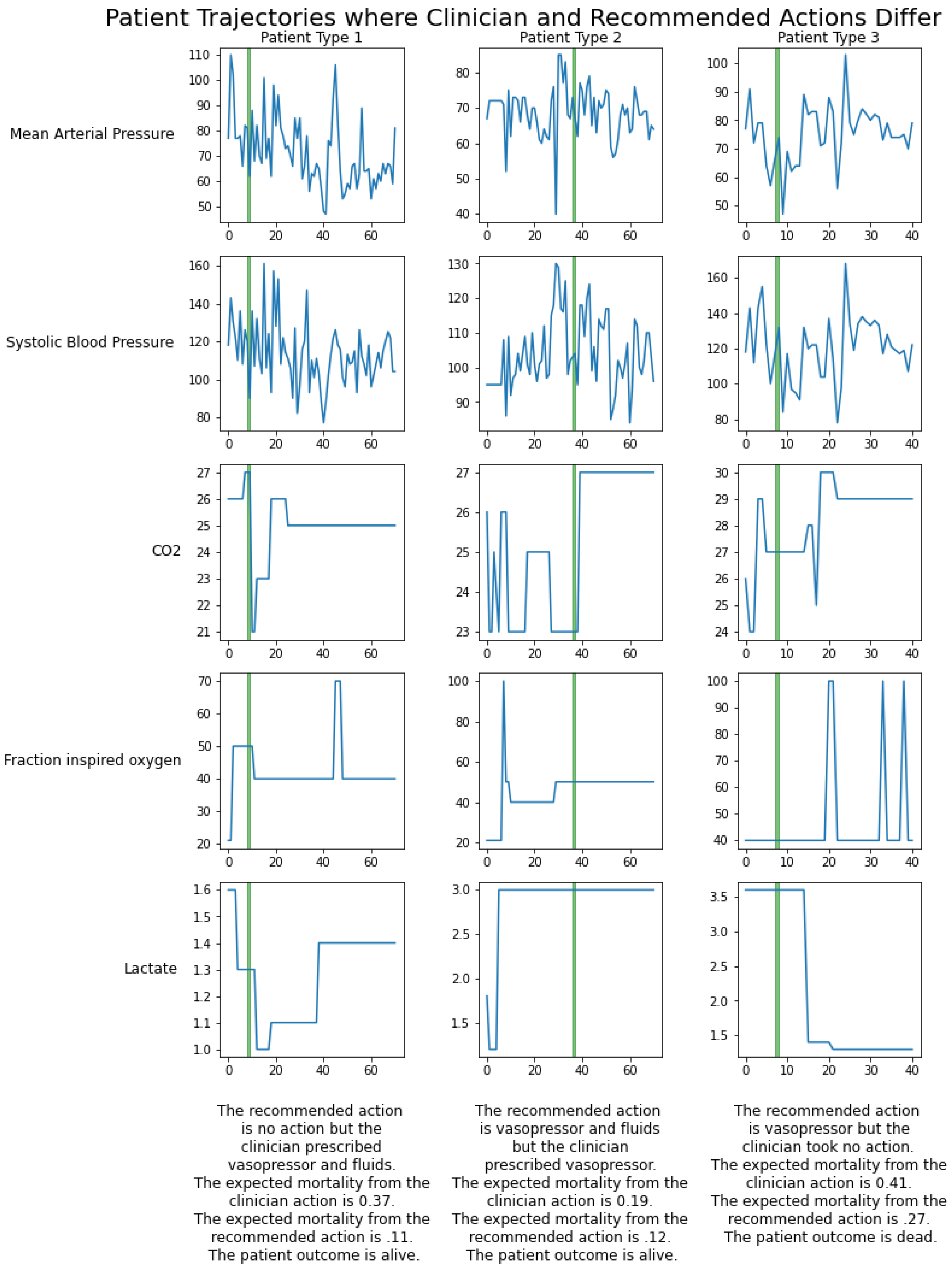

Supplementary Figure 3: **Patient Types where Recommended and Clinician Actions Differ.** Previous results. Three types of patients where the recommended action differs from the clinician actions.

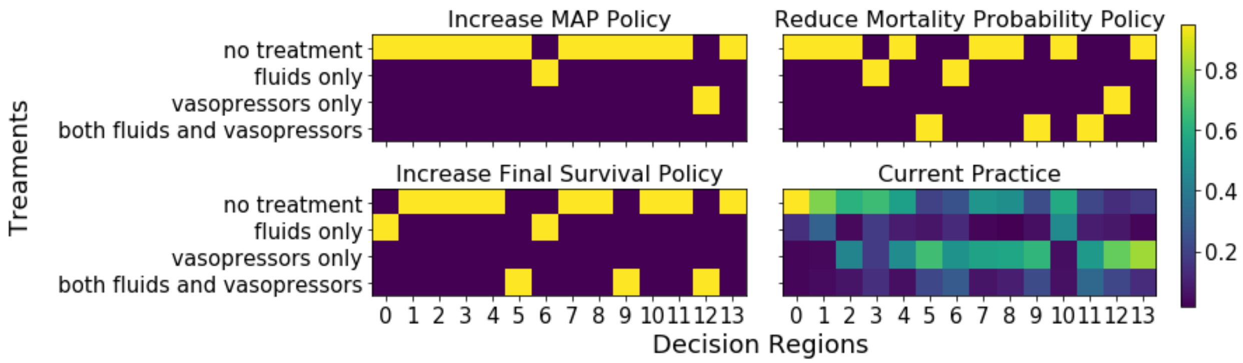

Supplementary Figure 4: **Policy Comparison.** Previous results. Probability assigned to each action under policies obtained using three different reward functions, compared to the current practices from clinicians.

Policy	WIS Score	ESS
Current Practice	-1.08 ±0.01	1,996 ±1
MAP-based Rewards	-0.94 ±0.01	994 ±16
Mortality-probability Rewards	-0.94 ±0.01	981 ±14
Final Survival-based Rewards	-0.93 ±0.02	996 ±20

Supplementary Table 1: **Off-policy evaluation results.** Previous results. Off-policy evaluation results of different policies across 5 bootstrap sample runs on the test set. MAP stands for Mean Arterial Pressure. More positive weighted importance sampling (WIS) score indicates better performance. Higher effective sample size (ESS) generally indicates a more reliable estimate.
